# A novel approach for predicting upstream regulators (PURE) that affect gene expression

**DOI:** 10.1038/s41598-023-41374-0

**Published:** 2023-10-30

**Authors:** Tuan-Minh Nguyen, Douglas B. Craig, Duc Tran, Tin Nguyen, Sorin Draghici

**Affiliations:** 1 Department of Computer Science, Wayne State University, Detroit, 48202 USA; 2 Department of Oncology, School of Medicine, Wayne State University, Detroit, MI 48201 USA; 3 Department of Computer Science and Software Engineering, Auburn University, Auburn, 36849 USA; 4 Advaita Bioinformatics, Ann Arbor, MI 48105 USA; 5 Department of Medicine, Washington University School of Medicine, St. Louis, MO 63110 USA

**Keywords:** Mechanism of action, Networks and systems biology, Computational biology and bioinformatics

## Abstract

External factors such as exposure to a chemical, drug, or toxicant (CDT), or conversely, the lack of certain chemicals can cause many diseases. The ability to identify such causal CDTs based on changes in the gene expression profile is extremely important in many studies. Furthermore, the ability to correctly infer CDTs that can revert the gene expression changes induced by a given disease phenotype is a crucial step in drug repurposing. We present an approach for Predicting Upstream REgulators (PURE) designed to tackle this challenge. PURE can correctly infer a CDT from the measured expression changes in a given phenotype, as well as correctly identify drugs that could revert disease-induced gene expression changes. We compared the proposed approach with four classical approaches as well as with the causal analysis used in Ingenuity Pathway Analysis (IPA) on 16 data sets (1 rat, 5 mouse, and 10 human data sets), involving 8 chemicals or drugs. We assessed the results based on the ability to correctly identify the CDT as indicated by its rank. We also considered the number of false positives, i.e. CDTs other than the correct CDT that were reported to be significant by each method. The proposed approach performed best in 11 out of the 16 experiments, reporting the correct CDT at the very top 7 times. IPA was the second best, reporting the correct CDT at the top 5 times, but was unable to identify the correct CDT at all in 5 out of the 16 experiments. The validation results showed that our approach, PURE, outperformed some of the most popular methods in the field. PURE could effectively infer the true CDTs responsible for the observed gene expression changes and could also be useful in drug repurposing applications.

## Introduction

Many life science experiments focus on comparisons between two phenotypes such as disease versus control, treated versus not treated, drug A versus drug B, etc. Microarrays and more recently, RNASeq assays, allow researchers to measure all genes and subsequently yield a list of differentially expressed (DE) genes. The challenge is to translate these measurements and lists of DE genes into a better understanding of the underlying biological phenomena, and in particular an understanding of its mechanisms. Analysis approaches such as pathway analysis^1–7^, network analysis^8^ and gene ontology (GO) analysis^1,9^, have been very successful in the past two decades in helping to translate such lists of DE genes into meaningful insights of the underlying biological phenomena. A particular sub-problem in this area focuses on the identification of upstream regulators that may explain the observed changes. In principle, such upstream regulators could be of different types including: genes (e.g. gene encoding transcription factors), miRNA, drugs, chemicals, or toxicants. This type of analysis is generally referred to as “causal analysis”^10–13^.

In some disease phenotypes, the presence of a chemical substance is responsible for the changes in the gene expression profiles and therefore, for creating the phenotypes. In other situations, a phenotype and its associated gene expression changes can be caused by the lack of a necessary chemical that plays an important metabolic role, e.g. iodine deficiency. Identifying the chemical, drug, or toxicant (CDT) that perturbs the patients’ gene expression level is a crucial step to pinpoint the cause and therefore help finding a suitable treatment for the patients.

Because understanding the effects of various CDTs is so important, the associations between chemicals and gene products have been studied intensely in the past decade and are available in several curated public chemical knowledge bases, such as the Comparative Toxicogenomics Database^14^, KEGG^15^, and Drugbank^16^.

Inferring the causal factors of the high-throughput gene expression profiles has been intensely researched and has become a helpful tool in: (i) elucidating and exploiting the mechanism of upstream regulators including upstream genes, proteins, or chemicals, and (ii) finding (alternative) treatments for studied conditions (e.g. drug repurposing). For example, Schadt et al.^10^ successfully identified three new genes in susceptibility to obesity by using this approach. Pollard et al.^17^ proposed a computational model to define the molecular causes of Type 2 Diabetes Mellitus. A Pfizer research group created networks of molecular causal interactions by integrating available biological knowledge, mainly from two commercial vendors: Selventa Inc. (http://www.selventa.com) and Ingenuity Inc. (http://www.ingenuity.com)^11^.

In this article, we describe a novel causal analysis tool for predicting upstream regulators (PURE) that can infer the cause of a set of measured gene expression changes. Given a set of differentially expressed genes between two phenotypes, PURE analyzes more than 5000 CDTs and their 330,659 known associations with human genome, 187,759 associations with mouse genome, and 161,323 associations with the rat genome as described in the Comparative Toxicogenomics Database^14^.

For each such CDT, PURE investigates two hypotheses: (i) that the gene expression changes are caused by the presence or overabundance of the given CDT and (ii) that the changes are caused by a deficiency of that CDT.

We assess the performance of PURE by comparing it with four classical methods, namely Over Representation Analysis using hypergeometric test^18^, Kolmogorov-Smirnov (KS)^19^, Wilcoxon^20^, FGSEA^21^, and a commercial tool, namely Ingenuity Pathway Analysis^12^. The result shows that our method outperforms existing methods in term of both the ability of identifying the causal CDT, as well as in terms of the number of false positives yielded by each method.

## Related work

Resources such as the Comparative Toxicogenomics Database, capture our collective existing knowledge about the genes that are affected by a multitude of chemicals, toxicants or drugs. This type of knowledge can be used for many purposes which in turn can generally be categorized into two main directions: drug repositioning and causal analysis.

The goal of *drug repositioning* is to identify new therapeutic applications for existing drugs. Since these drugs are already approved, they can skip the Phase I clinical trials in the drug approval pipeline, i.e. testing the safety of the drug. Therefore this approach is faster and more cost-efficient than the process of new drug discovery, which takes on average 15 years and more than one billion dollars for each drug^22^. Most often the gene profiling of control samples and treated samples are compared to obtain a list of DE genes, also defined in some literatures as the summary compound’s effect^23^. There are two popular approaches for *in silico* drug repositioning: these summary compound’s effect are compared to a disease-associated DE genes which obtained by the contrast between healthy and disease samples’ gene expression; or to other compound’s effect^24^. The former approaches hypothesize that if the compound’s effect are negatively correlated with the disease DE genes, e.g. a gene is up-regulated by a drug’s effect and down-regulated by a disease, that compound would be a good candidate to revert the phenotype’s DE genes, and hence can potentially suppress the phenotype^25,26^. Some examples of studies using this idea in drug repurposing: Claerhout et al. proposed using vorinostat as a candidate treatment for gastric cancer^27^; Chen et al.^28^ successfully identified and verified chlorpromazine and trifluoperazine as the alternative for sorafenib to treat hepatocellular carcinoma; Dudley et al.^29^ proposed topiramate which was approved for epilepsy as a alternative treatment for inflammatory bowel disease. Methods in the latter approach work under an assumption that if two drugs evoke similar summary compound’s effects, they could share a common mode of action^30–32,32–34^. The majority of the methods in both groups utilize Connectivity Map (cMap)^35,36^ as reference of signature of differential gene expressions of disease and drug responses.

*Causal analysis* refers to an analysis that aims to infer the CDT that potentially causes the observed expression changes. The methods in this category often hypothesize that a drug compound could cause a disease phenotype if the compound’s gene signature is positively correlated with disease’s gene signature^37^. Although this approach uses the gene expression profiles to reach the same goal as our proposed method, it utilizes a totally different technique. A more direct approach to identify the CDT is applying graph theories on the cause-effect network between CDT and genes. Chindelevitch et al. used two commercial knowledge bases, Selventa Inc. (http://www.selventa.com) and Ingenuity Inc. (http://www.ingenuity.com) to construct a network of molecular causal interactions that would suggest molecular hypotheses that explain the observed changes in gene expression profiles. For each molecule, they used a scoring system which performs a subtraction of the number of genes against the hypotheses from the number of genes supporting the hypotheses^11^. Subsequently, they applied the distribution of the scores under the null and Fisher’s exact test to compute the statistical significance. More recently, Krämer et al. published a paper that presents the causal analysis approach in Ingenuity Pathway Analysis (IPA). This work has very similar goals with our approach, hence we will discuss and compare its performance with ours in the following sections. Although there are computational methods using the similar techniques on specific applications, large-scale and more general attempts are scarce in this field.

## Methods

### Knowledge base

First, we preprocess the network of drug-gene interactions from the Comparative Toxicogenomics Database^14^ that provides manually curated information about associations between more than 5000 CDTs and 10,000 of genes from many species including human, mouse, rat, etc. These data include the chemical family, the CDT-gene, and CDT-disease relationships. There are various types of relationships between a CDT and targeted or affected genes, such as increase/decrease expression, increase/decrease abundance, or increase/decrease methylation. In this analysis, since our goal is to analyze gene expression measurements, we focus on those effects leading to an expression increase or decrease. Henceforth, these will be referred to as “activation” and “inhibition” effects.

### Data sets

We downloaded 16 benchmark data sets from Gene Expression Omnibus database (GEO: https://www.ncbi.nlm.nih.gov/geo/). These experiments varied from human, mouse, to rat with 8 different CDTs (See Table 2).

The DE genes are selected using a threshold of $|log(FC)| > 0.6$ and *p* value $< 0.05$.

### Two hypotheses

For each CDT, we consider two hypotheses:

- Hypothesis 1 (H1): The level of the CDT is higher in the phenotype compared to the control.
- Hypothesis 2 (H2): The level of the CDT is lower in the phenotype compared to the control (or completely absent).

### Statistical significance

Let $G_{DE}$ be the set of differentially expressed genes available in the gene expression profile; $C_{KB}$ be the set of all CDTs in the knowledge base (KB); $G_{KB}$ be the set of genes in the KB, and $E_{KB}$ be the set of edges that represents the associations between CDTs and genes in the KB.

**Figure 1:**
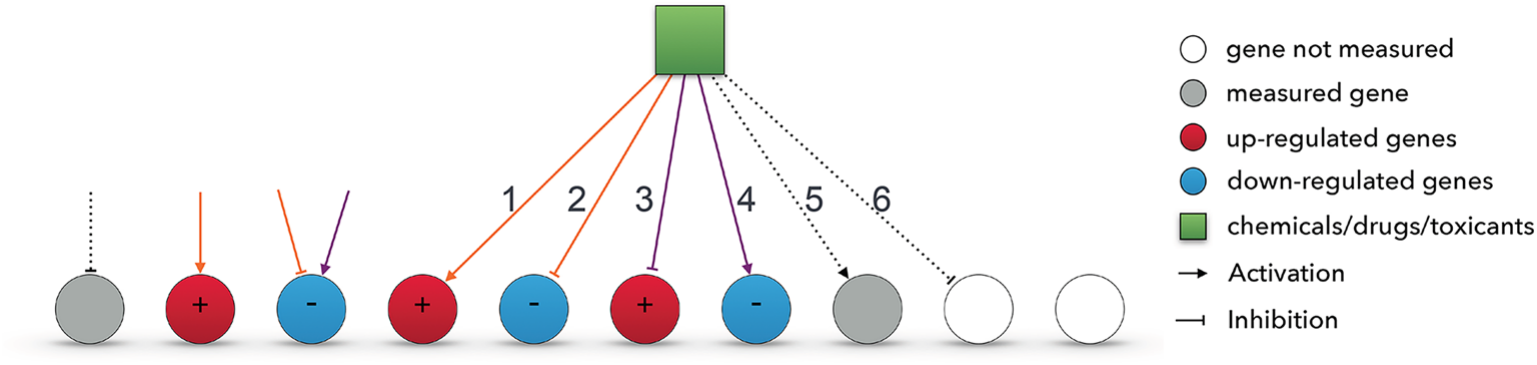
Illustration of different types of genes available in the Comparative Toxicogenomics Database and gene expression profile, and their relationships with the chemical. The significance of a chemical/drug/toxicant (green box) in each hypothesis is assessed using a statistical test based on the number of up- or down-regulated DE genes and their associations with the drug. The orange edges (e.g. 1 and 2) are the ones that support the hypothesis that level of studied chemical/drug/toxicant is higher in the phenotype compared to the control while the purple ones (e.g. 3 and 4) are supporting the hypothesis that its level is lower compared to the control.

We define G as the set of genes included in both $G_{DE}$ and $G_{KB}$, i.e. $G_{DE} \cap G_{KB}$. Subsequently, $C \subseteq C_{KB}$ and $E \subseteq E_{KB}$ represent the set of all CDTs and their corresponding associations with those genes in *G* available in the knowledge base. These sets are formally defined as follows:

1 $$\begin{aligned} C = \{c \in C_{KB} \mid \exists g \in G \wedge \exists e_{c,g} \in E_{KB} \} \end{aligned}$$

and

2 $$\begin{aligned} E = \{ e_{c,g} \mid \exists c \in C \wedge \exists g \in G \ \wedge e_{c,g} \in E_{KB} \} \end{aligned}$$

where $e_{c,g}$ denotes an edge from an upstream CDTs *c* to targeted gene *g* representing their relationship described in the Comparative Toxicogenomics Database. The sign of the edge, $s(e_{c,g})$, reflects the type of the association, namely positive ($+$) for an activation and negative (−) for an inhibition edge. Also, we denote *s*(*g*) the sign of the DE gene *g* which is positive if *g* is up-regulated, and negative, otherwise.

Each edge $e \in E$ is labeled whether it is supporting either of the testing hypotheses (Fig. 1). For example, if an edge is activation and its targeted DE gene is indeed up-regulated, the edge supports the hypothesis 1. In essence, an edge is considered to be supporting the hypothesis 1 when its sign, $s(e_{c,x})$, is aligned with the sign of its targeted DE gene, namely $s(e_{c,g}) = s(g)$. Such edges are colored orange in Fig. 1. Edges whose signs are opposite with the expression direction of their targeted DE genes are considered as a supporting evidence for the hypothesis 2, and are colored purple in Fig. 1. Notice that $E^{H1}$ and $E^{H2}$ are the two mutually exclusive sets of edges that support the first and second hypothesis, respectively, because an edge $e \in E$ must support either the hypothesis 1 or hypothesis 2, but not both. Formally, $E^{H1} \cap E^{H2} = \emptyset$ and $E^{H1} \cup E^{H2} = E$.

For each chemical $c \in C$, a statistical score, i.e. *p* value, for each aforementioned hypothesis is then computed using the one-sided Fisher’s exact test. Without losing generality, let us discuss the hypothesis 1. First, a confusion matrix is constructed as in Table 1, where *l*, *k*, *m*, and *n* are the number of edges related to *c* that support the hypothesis, the number of edges related to *c* that do not support the hypothesis, the number of edges not related to *c* that support the hypothesis, and the number of edges not related to *c* that do not support the hypothesis, respectively. The probability of this observed contingency under the null hypothesis is defined as follows:

3 $$\begin{aligned} p = \frac{ {l+k \atopwithdelims ()l}{m+n \atopwithdelims ()m}}{{\Vert E\Vert \atopwithdelims ()l + m}} \end{aligned}$$

where $\Vert E\Vert = (l+k+m+n)$ is the number all edges in *E*.

The *p* value of the one-sided Fisher’s exact test is the sum of the probabilities of all contingency tables that have the number of edges supporting the H1 more than *l* where the number of edges related to the compound *c* and the number of edges supporting the H1 are unchanged.

Notice that because an edge supporting H1 is against H2 and vice versa, the contingency matrix for H2 can be obtained by swapping the columns of the observed contingency matrix of H1. Hence, the *p* value for the H2 can also be derived from these four numbers.

Finally, we use the false discovery rate (FDR) to correct the *p* values for multiple comparisons^38^.

**Table 1:** For each chemical/drug/toxicant *c*, a contingency table is constructed.

	Supporting H1	Against H1
Related to *c*	*l*	*k*
Not related to *c*	*m*	*n*

Here, *l*, *k*, *m*, and *n* are the number of edges coming from *c* that support the hypothesis 1 (H1), the number of edges coming from *c* that do not support the H1, the number of edges not coming from *c* that support the H1, and the number of edges not coming from *c* that do not support the H1, respectively. Note that the edges that support H1 are against the hypothesis 2 (H2), and vice versa. Hence, a similar contingency table for H2 can be constructed using these four numbers.

## Results

We evaluate and compare the performance of our proposed method with the other five approaches, namely Over Representation Analysis (ORA) using hypergeometric test, Kolmogorov-Smirnov test (KS)^19^, Wilcoxon^20^, FGSEA^21^, and the causal analysis used in Ingenuity Pathway Analysis (IPA)^12^.

### Benchmarking methods

The ORA family of methods, as well as other tests, such as KS, Wilcoxon, and FGSEA, are widely used in gene set analysis to determine whether a particular gene set—such as the genes associated to a given GO term or pathway—is significantly affected in the given phenotype. In principle, the same approach could be used to decide whether a given CDT could be related to the phenotype by considering the set of genes known to be affected by the given CDT.

*ORA* uses a statistical test, such as hypergeometric, chi-square, or binomial distribution, to evaluate if the number of DE genes is over- or under-represented in the set of targeted genes of a CDT. In this study, we use hypergeometric test to compute *p* value, namely the probability of getting the *x* or more observed DE genes in *M* CDT’s downstream genes from a pool of *N* background genes with *n* DE genes. Mathematically, this probability is defined as:

$$\begin{aligned} P(X \ge x) = 1-P (X \le x -1)= 1- \sum _{i=0}^{x-1} \frac{\left( {\begin{array}{c}M\\ i\end{array}}\right) \left( {\begin{array}{c}N-M\\ n-i\end{array}}\right) }{\left( {\begin{array}{c}N\\ n\end{array}}\right) } \end{aligned}$$

ORA does not take into consideration if the DE gene is up-regulated or down-regulated, nor the type of associations between the CDT and the targeted DE gene.

The Kolmogorov-Smirnov test (KS) determines whether there is a significantly difference between two empirical distributions of the scores (the direction of the fold changes) of the DE genes targeted by the CDT (DEhit) and those of the DE genes not targeted by the CDT (DEmiss). First, all genes will be ranked in order of their log fold changes. Then, we calculate the cumulative distribution function (CDF) of the ranked gene list for background genes and for the genes affected by the CDT. Finally, the KS test is used to compare the two CDFs and calculates a *p* value that measures the significance of the enrichment. Although KS takes the sign of the DE genes into consideration (the fold changes of gene expression), it ignores the type of associations between CDTs and DE genes.

*Wilcoxon* is a rank-based non-parametric test for comparing the ranks of DE genes affected by the CDT (DEhit), and other DE genes (DEmiss). First, it ranks all genes in both lists based on their fold changes. Subsequently, it computes the a test statistic *W*, which is the sum of the ranks for all DEhit. This test statistic *W* is compared to the distribution of *W* under the null hypothesis. The null hypothesis is rejected if *W* is extreme and falls outside 95% of the distribution. In R, the Wilcoxon is available via the function wilcox.test. Similar to ORA, the associations of the affected DE genes with the CDT are completely ignored.

*FGSEA* is an improvement of the Gene Set Enrichment Analysis (GSEA) approach. FGSEA accelerates the calculation of the GSEA *p* value by estimating it with a high accuracy (the estimation error is less than $10^{-100}$ when compared with actual GSEA *p* value). GSEA, in turn, is one of the most popular approaches in gene set analysis^39^. It consists of three important steps: computing the enrichment score for each gene set, estimating the statistical significance of the enrichment score, and adjusting for multiple hypothesis testing. We used the function fgsea in the “fgsea” package with the parameters nperm = $10^{4}$ and minSize = 15.

*IPA* is a commercial web-based platform that offers several applications including a causal analysis tool^12^. Given a list of DE genes, IPA’s causal analysis outputs a list of upstream regulators including chemicals/drugs, as well as genes, proteins families, complexes, microRNA, and biological processes. Notice that including all these types of regulators in the report would worsen the IPA’s result when benchmarking with other methods since it might increase the rank of the true CDT. Moreover, since there is only one true causal CDT in each experiment and all other elements are considered as false positives, having them in the result would increase the number of false positives. For these reasons, beside the default IPA result, we added to the method benchmarking the so-called IPA-CDT that only retains the CDTs in the IPA report and excludes all non-chemical elements.

According to Krämer et al., IPA derives two scores for each regulator *r*, namely the overlap *p* value and the activation z-score, as follows.

*The overlap p value* reflects the enrichment of the list of *r*-regulated genes in the set of all DE genes without taking the regulation direction into consideration. Formally, it is based on the one-sided Fisher’s exact test and is calculated as follows:

$$\begin{aligned} {p(r)=\sum _{k = 0}^{min(c,d)} \frac{(a+b)!(c+d)!(a+c)!(b+d)!}{(a+k)!(b-k)!(c-k)!(d+k)!n!}} \end{aligned}$$

where *n* is the number of all background genes, i.e. all the genes in the data set that have at least one association with any upstream regulator, *a* is the number of DE genes regulated by *r*, *b* is the number of DE genes that are not regulated by *r*, *c* is the number of *r*-regulated genes but not differentially expressed, and $d=n-a-b-c$.

*The activation z-score* uses the information about the direction of gene regulation to predict the regulators. Let ${\tilde{O}}$ be the set of *r*-regulated DE genes, the activation z-score of the corresponding regulator *r* is defined as:

$$\begin{aligned} { z(r) = \frac{\sum \limits _{v \in {\tilde{O}}}w_{R}(r,v) s_{R}(r,v) s_{D}(v)}{\left( \sum \limits _{v \in {\tilde{O}}}[w_{R}(r,v)]^2\right) ^{1/2}}} \end{aligned}$$

where $w_{R}(r,v)$ represents the weight associated with the regulation of *r* and the downstream DE gene *v*, $s_{R}(r,v)$ is the sign of the regulation, i.e. $s_{R}(r,v)=1$ for activation and $s_{R}(r,v)=-1$ for inhibition, and $s_{D}(v)$ represents the direction of DE gene’s expression, i.e. $s_{D}(v)=1$ for up-regulation and $s_{D}(v)=-1$ for down-regulation, respectively. The activation z-score is proven to be approximately normally distributed under the null model, e.g. random signs $s_{R}(r,v)$ and $s_{D}(v)$. On one hand, a high positive z-score, e.g. $z(r) > 2$, indicates that the match between the signs of downstream DE genes ($s_{D}(v)$) and the corresponding edges ($s_{R}(r,v)$) is significant, which in turn suggests that *r* is the activated regulator. On the other hand, a low negative z-score, e.g. $z(r) < -2$, is an indicator that the sign of the downstream DE genes are mostly opposite with the corresponding regulations. In this case, *r* is predicted as an inhibitor^12^.

Among all the benchmarking methods included in this study, only IPA takes the sign of CDTs—genes associations under consideration and can predict whether a significant CDTs is activated or inhibited (corresponding to H1 and H2), as PURE does, so a more detailed theoretical comparison is warranted. Although IPA derives these two scores described above for each CDT, the result is solely determined by the z-score: the regulator is determined as “activated” or “inhibited” if its z-score $\ge 2$ or $\le -2$, respectively^12^. In other words, the statistic calculated from the data will determine the outcome. In contrast, PURE uses a more classical approach in which the hypotheses are formulated before hand, independently of the data as in a canonical hypothesis testing. PURE considers each hypothesis separately, and calculates a p-value that will indicate whether the null hypothesis can be rejected. For PURE, the null hypothesis is that “CDT X has not had an impact on the measured gene expression changes” whereas the first research hypothesis is that “CTD X was present and had an impact on the gene expression changes” and the second, independent, research hypothesis is that “CTD X was lacking and its absence had an impact on the gene expression changes”. The testing done in the proposed approach is more rigorous in terms of statistical testing, but such approach can potentially reject the null hypothesis for both research hypotheses which would be difficult to interpret from a biological perspective. In contrast, the approach used by IPA avoids such potentially ambiguous situations because the z-score can be either positive or negative but not both. The most important difference stemming from these two approaches is that PURE can identify CDTs that can reverse the observed genes expression changes because it considers both sets of statistical hypothesis. This means that PURE can be used for drug repurposing - situations in which one is given a gene expression profile associated with a given disease and the task is to identify a drug that could revert some of the changes. In contrast, IPA only considers the CDTs that are present and focuses whether they are “activated” or “inhibited”. Another difference worth mentioning between IPA and PURE is that while PURE considers and derives a *p* value corresponding to the hypothesis testing for every CDT in the knowledge base, IPA does not derive z-score for all CDT in the knowledge base. For example, Methylprednisolone is in the IPA’s knowledge base and has z-score in the experiment 15, but no z-score is reported in the experiment 16 (Table 5).

### Testing Hypothesis 1

We evaluate these methods using 14 benchmark data sets from three different species, namely human, mouse, and rat (See Table 2). In these data sets, gene expressions were measured after the ingestion of a given CDT. Hence, the cause of all the changes observed throughout the system is known. Furthermore, this particular situation corresponds to H1, where the level of the CDT is higher than normal. We consider the administered CDT as the “true CDT” for each of these data sets.

The result of each method is a ranked list of CDTs based on the particular statistic used by each method, i.e. FDR-adjusted *p* values for PURE, ORA, KS, Wilcoxon, and FGSEA, and z-score for IPA and IPA-CDT. If several CDTs are ranked with the same statistic, we use an average. For instance, if the top 4 elements have the same *p* value, they would be all ranked as 2.5 instead of 1, 2, 3, and 4, respectively because 2.5 is the average of the set $\{1, 2, 3, 4\}$. An ideal method would be able to identify the true CDT by ranking it on top with a significant *p* value $\le 0.05$ or z-score $\ge 2$ (or z-score $\le -2$ in case of IPA testing the H2).

**Figure 2:**
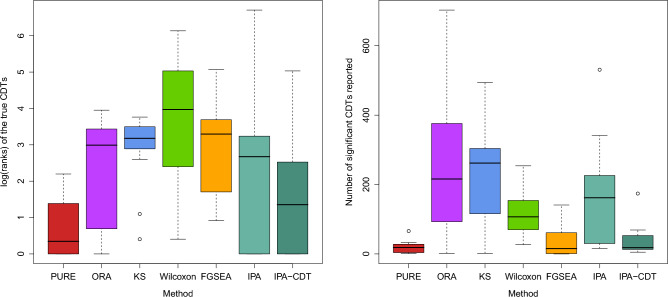
The comparison of PURE and other 5 methods, in term of log(ranks) of true CDTs (left panel) and the number of significant chemicals reported (right panel). In the left panel, a better method would rank the true CDT as low as possible (ideally rank it 1) so lower log(rank) values are better. A CDT that is different from the true CDT and yet reported as significant is a false positive. For this reason, we would like the number of CDTs reported as significant (shown in the right panel) to also be as low as possible.

There are 14 data sets corresponding to the H1 where the causal CDTs are known (Table 2). We report and compare the ranks of the true CDTs in these 14 data sets (see Table 3 and Fig. 2a). PURE (average = 2.8) is better than all of the methods in this study, followed by IPA-CDT (average = 17.7), ORA (average = 20.1), and KS (average = 24), GSEA (average = 32.9), IPA (average = 79.9), and Wilcoxon (average = 104.4) (Table 3). PURE can successfully identify the true CDTs in these 14 benchmarking data sets and rank them at the top 7 times. It performs better than all of the methods in 9 data sets. IPA-CDT performs best in 6 data sets (3 of which are tied with PURE) and is able to rank the true CDTs at the top 5 times. However, it cannot identify the true CDTs in three data sets, in two of which the true CDTs are not present in the result list (data set 8 and data set 14). Similar to IPA-CDT, IPA can correctly rank the true CDTs at the top in 5 data sets. FGSEA performs best in 2 data sets. ORA performs best in one data sets (tied with PURE), while KS and Wilcoxon are not able to perform best in any of the experiment.

However, an evaluation based solely on the method’s ability to identify the true CDT using the *p* value does not show the whole story and sometimes misleads. For example, a method that derives low *p* values for all CDTs can always identify the true CDT, but is still considered a bad one because it includes a lot of false positives in the result. Therefore, we also take the number of false positives in the result into consideration, i.e. the number of CDTs that are not true CDT but reported as significant (Fig. 2b). Although chemicals and drugs could have similar effects or could be in the same family, we only consider the true CDT as the one and only true positive and all other CDTs as true negatives. We expect a good method would derive a low number of CDTs in the result, ideally only one, the true CDT. Our method generally reports the lower numbers of reported chemicals (average = 19.4) than any other methods compared, followed by FGSEA (average = 37.4) and IPA-CDT (average = 37.6). Although FGSEA is comparable to IPA-CDT, it cannot identify the true CDTs in 6 out of 14 experiments while IPA cannot identify only 3 out of 15. Wilcoxon and IPA report on average more than 100 CDTs while ORA and KS report on average more than 200 CDTs per experiment (Table 4).

To investigate whether or not PURE is superior to the other methods, we used a Wilcoxon test to compare the ranks and number of CDTs reported by PURE with those provided by the other methods. The *p* values for the rank comparison of PURE and IPA-CDT, ORA, KS, FGSEA, IPA, and Wilcoxon are 0.02, 4E−4, 8E−6, 4E−5, 6E−3, and 1E−5, respectively. Notice that in some experiments, the ranks of the true CDTs are not available (e.g. IPA in the experiment 9). In these cases, we replace the NA ranks by the number of significant CDTs reported in the corresponding experiment plus one, e.g in the experiment 9 performed by IPA, we assigned 31 to the true CDT’s rank because IPA reported a list of 30 significant CDTs but the true CDTs is not included (Table 4). The *p* value for the number of CDTs reported comparison between PURE and IPA-CDT, ORA, KS, FGSEA, IPA, and Wilcoxon are 0.04, 4E−4, 9E−6, 0.4, 4E−5, and 2E−6, respectively. Since all *p* values are less than the standard threshold of 0.05 (except for FGSEA while comparing the number of CDTs reported), PURE’s performance can be considered significantly better than all of the methods included in the study, in terms of both the rank of the true CDTs, as well as the number of significant CDTs reported.

### Testing Hypothesis 2

The assessment of these methods on testing H2 is more challenging since the data sets in which the ground truth is known are scant, i.e. an CDT is truly lacking in the system. Another important application for testing the H2 is drug repurposing. A CDT could potentially reverse the gene expression changes caused by the disease and therefore be a candidate for drug repurposing. At the time we applied PURE to the COVID-19 data, the recommendation of several organizations, including the World Health Organization (WHO), CDC, and Surviving Sepsis Campaign, was against the use of any systemic corticosteroids in the severe cases of COVID-19^40^. Surprisingly, our results showed that Methylprednisolone, a corticosteroid, would be effective in helping the patients with severe disease^41^. Subsequently, ours and other’s clinical trials have shown that indeed steroids are effective and the world health organization has reversed their recommendation^42–46^. At this time, the standard of care in severe cases of COVID-19 is the corticosteroid treatment. Hence, in this study, we use the data set GSE147507 in which the expression of NHBE and A549 cells infected with COVID-19 were compared with their corresponding control, and consider Methylprednisolone as the “target” CDT in the experiments 15 and 16 for different contrasts (Table 5).

We evaluate the methods’ performance based on the same criteria: rank of the target CDT (Methylprednisolone), and the number of CDTs reported. Our proposed method, PURE, is able to identify Methylprednisolone in both experiments with the average rank of 3.25, followed by FGSEA (average = 6.5), ORA (average = 14.75), KS (average = 19.5), Wilcoxon (average = 29), IPA-CDT (average = 393), and IPA (average = 890). Notice that although ORA, KS, Wilcoxon, and FGSEA can identify Methylprednisolone as significant CDT, they cannot determine whether it is present or absent. Also, IPA cannot identify Methylprednisolone in either experiments. More importantly, all other methods, except for FGSEA, reported hundreds of significant CDTs in each experiment, which make it difficult for a researcher to identify a truly effective drug such as Methylprednisolone. Hence, in term of number of CDTs reported, PURE also performs better than other methods. The average number of CDTs reported by PURE is 10 CDTs, whereas that number of FGSEA, IPA-CDT, Wilcoxon, KS, IPA, ORA are 27.5, 103, 189, 216, 238, 553, respectively (Table 5). Since the sample size is small (only 2 experiments), we do not compute *p* values for these comparisons.

**Table 2:** The detailed information of 16 benchmarking data sets used in this manuscript.

Experiment ID	GEO ID	Organism	True CDT	Hypothesis
1	GSE26487^47^	Human	Dexamethasone	H1
2	GSE49804^48^	Mouse	Dexamethasone	H1
3	GSE86837^49^	Mouse	Diethylhexyl Phthalate	H1
4	GSE58434_H^50^^a^	Human	Calcitriol (Vitamin D)	H1
5	GSE58434_Ast^50^^b^	Human	Calcitriol (Vitamin D)	H1
6	GSE11352_12h^51^	Human	Estradiol	H1
7	GSE11352_24h^51^	Human	Estradiol	H1
8	GSE11352_48h^51^	Human	Estradiol	H1
9	GSE74000^52^	Human	Acetaminophen	H1
10	GSE12446^53^	Human	Estradiol	H1
11	GSE67266_WT^c^	Mouse	Etoposide	H1
12	GSE67266_KO^d^	Mouse	Etoposide	H1
13	GSE51213	Mouse	Dexamethasone	H1
14	GSE58875^54^	Rat	Copper deficiency	H1
15	GSE147507_NHBE^55^^e^	Human	Methylprednisolone	H2
16	GSE147507_A549^55^^f^	Human	Methylprednisolone	H2

All data sets are downloaded from Gene Expression Omnibus (GEO) database.

^a^ Contrast: Healthy patient treated with vitamin D versus healthy patient untreated.

^b^ Contrast: Asthma patient treated with vitamin D versus asthma patient untreated.

^c^ Contrast: Wild Type (WT) mice treated with etoposide versus mock treated after 6 h.

^d^ Contrast: MK2/3 knockout (KO) mice treated with etoposide versus mock treated after 6 h.

^e^ Contrast: Primary normal human bronchial epithelial cells (NHBE) infected with COVID-19 versus control.

^f^ Contrast: A549 lung cell line infected with COVID-19 versus control.

**Table 3:** Benchmarking the methods in term of the ranks of the true CDTs.

Exp. ID	GEO ID	Organism	Rank of true CDTs						
			PURE	ORA	KS	Wilcoxon	FGSEA	IPA	IPA-CDT
1	GSE26487	Human	**1**	1.5	43	45	25.5	**1**	**1**
2	GSE49804	Mouse	2	2	33	11	16.5	**1**	**1**
3	GSE86837	Mouse	**1**	**1**	3	2.5	47	816	153
4	GSE58434_H	Human	**1**	16	13.5	65	159	25	14
5	GSE58434_Ast	Human	**2**	18	23.5	254	30.5	26	5
6	GSE11352_12h	Human	**1**	32.5	24.5	126	4.5	**1**	**1**
7	GSE11352_24h	Human	**1**	33	24	214	7.5	**1**	**1**
8	GSE11352_48h	Human	2	29.5	24	153	24.5	**1**	**1**
9	GSE74000	Human	**1**	16	23	9	75	*NA*	*NA*
10	GSE12446_WT	Human	4	31	39.5	460.5	27.5	10	**3**
11	GSE67266_KO	Mouse	6	22	27	32	**3.5**	22	12
12	GSE67266	Mouse	7.5	25	38	62.5	**2.5**	21	7
13	GSE51213	Mouse	**9**	52	18	26	33	34	13
14	GSE58875	Rat	**1**	1.5	1.5	1.5	3	*NA*	*NA*
Average ± std. dev.			**2.8 ** ± **2.7**	20.1 ± 15.2	24.0 ± 12.3	104.5 ± 130.4	32.8 ± 41.5	79.9 ± 232.1	17.7 ± 42.9

The experiment IDs (Exp. ID) are corresponding to the ones in Table 2. The lower the rank of the true CDTs, the better. The Bold highlighted cell is the best one in each experiment. PURE performs best in 9 out of 14 experiments, followed by IPA which performs best in 6 experiments (3 co-best with PURE). In two of the data sets analyzed IPA was not able to identify the correct CDT at all (highlighted in Italic).

**Table 4:** Benchmarking the methods in term of the number of significant CDTs reported.

Exp. ID	GEO ID	Organism	Number of significant CDTs reported						
			PURE	ORA	KS	Wilcoxon	FGSEA	IPA	IPA-CDT
1	GSE26487	Human	**1**	58	117	45	*2*	23	***14***
2	GSE49804	Mouse	**4**	***14***	67	47	*0*	***18***	***10***
3	GSE86837	Mouse	**1**	93	104	77	*0*	*191*	*14*
4	GSE58434_H	Human	***13***	703	257	167	*89*	170	75
5	GSE58434_Ast	Human	**8**	520	267	*154*	*19*	199	35
6	GSE11352_12h	Human	28	376	299	*123*	***18***	79	**6**
7	GSE11352_24h	Human	25	384	325	*154*	32	125	***13***
8	GSE11352_48h	Human	31	369	304	*131*	62	324	22
9	GSE74000	Human	***17***	248	300	82	*7*	*30*	*14*
10	GSE12446_WT	Human	33	297	494	*236*	139	226	31
11	GSE67266_KO	Mouse	***20***	118	116	70	***10***	156	61
12	GSE67266	Mouse	22	96	118	91	***13***	341	53
13	GSE51213	Mouse	66	184	331	254	133	533	174
14	GSE58875	Rat	**2**	**2**	**2**	27	*0*	*16*	*5*
Average ± std. dev.			19.4 ± 17.5	247.3 ± 206.1	221.5 ± 135.3	118.4 ± 69.2	37.4 ± 49	173.6 ± 148.7	37.6 ± 44.9

The experiment IDs (Exp. ID) are corresponding to the ones in Table 2. The cell is highlighted Bold if the number of reported CDTs less than 10; Bold Italic if it is more than or equal to 10 but less than or equal 20. The cell is highlighted Italic if the true CDT is not included at all in the reported list of significant CDTs by the method (i.e. all reported CDTs are false positives). For instance, in the first row PURE only reported only one CDT and that was the correct one (zero false positives). Hence, PURE’s cell is highlighted Bold. FGSEA reported two significant CDTs but the cell is highlighted Italic because these reported CDTs are false positives. The true CDT was not significant and was ranked 25.5 (Table 3). In the same data set, IPA-CDT ranked the true CDT first (Table 3). However, the cell is highlighted Bold Italic because it also included 13 other CDTs which are considered false positives.

**Table 5:** Benchmarking the methods for accepting the H2.

Exp. ID	GEO ID	Organism	Rank of true CDTs						
			PURE	ORA	KS	Wilcoxon	FGSEA	IPA	IPA-CDT
15	GSE147507_NHBE	Human	**2.5**	23	12	24	8.5	*890*	* 393*
16	GSE147507_A549	Human	**4**	6.5	27	34	4.5	*NA*	*NA*
Average			**3.25**	14.75	19.5	29	6.5	890	393

Exp. ID	GEO ID	Organism	Number of significant CDTs reported						
			PURE	ORA	KS	Wilcoxon	FGSEA	IPA	IPA-CDT
15	GSE147507_NHBE	Human	**8**	719	274	224	40	*371*	*182*
16	GSE147507_A549	Human	**12**	387	159	154	15	*105*	*24*
Average			**10**	553	216	189	27.5	238	103

Bold highlighted cells are best for each row. Italic highlighted cells indicate that the target CDTs are not included in the reported list. Notice that ORA, KS, Wilcoxon, and FGSEA reject the null hypothesis and identify the target CDTs as significant to the observed changes in the gene expression profiles, they do not distinguish the two hypotheses H1 and H2. Beside PURE and FGSEA, all other methods include more than hundred CDTs in the significant lists.

## Discussion

In this manuscript, we propose PURE, a causal analysis approach that infers the CDTs responsible for the changes in the gene expression profile, either because their level in the subject’s system is higher (hypothesis 1) or lower (hypothesis 2) than normal. On one hand, hypothesis 1 helps identifying the cause of conditions related to external CDTs, and therefore is a crucial step for the treatment process. PURE can be applied to time series analysis experiments in which the subjects’ gene expression profiles are measured periodically after taking a known medicine. Applying our method on these profiles can identify the time point beyond which the effect of the drug ceases to affect the gene expression profiles in a significant way (e.g. experiments 6–8 in Table 2). On the other hand, hypothesis 2 can be interpreted as a prediction of what is lacking in the system but also as a suggestion for a CDT that could reverse the expression changes induced by a disease phenotype, and therefore, is useful in the drug repurposing study. Recently, a similar approach was applied to the discovery of a treatment for severe cases of COVID-19^41^.

The existing approaches in the field are limited in either one of the following ways: (i) they focus on identifying upstream genes/proteins instead of external causes such as CDTs, (ii) they do not take the type of the interactions between chemical and genes into consideration, and (iii) they only focus on some specific conditions. PURE addresses all of those shortcomings.

In the validation process, we tested the proposed method on H1 with 14 gene expression data sets with different known causal factors and different species and on H2 with other 2 data sets. The results show that our method is more robust than other classical and commercial approaches included in this study, in term of the rank of true CDT, and the number of false positives.

There are several reasons that limit the accuracy of the existing tools compared to PURE. First, they do not take into consideration the direction of changes in DE genes, i.e., current methods do not take into consideration if a DE gene is up- or down-regulated. Second, they do not utilize the information about CDT-gene interactions as PURE does. Finally, Fisher’s exact test may not yield reliable when the number of “interesting” genes (i.e. DE genes) is small, which is often the case in gene expression data sets. Instead of DE genes versus total number of genes, PURE considers the number of interactions supporting (or not) the testing hypothesis. Because the ratio of edges supporting H1 out of all edges is much higher than the ratio of DE genes out of all available genes in the data sets, PURE is expected produce a significantly more accurate result in more situations.

### Limitations

The proposed method might be useful in many cases but only as a first—*computational*—step to identify potential causal CDTs (by using H1), or drugs that could be potentially repurposed (by using H2). As any other type of computational, *in silico* results, anything obtained with this approach will require further validation through laboratory experiments, clinical trials or both.

The proposed approach, as well as all other methods benchmarked in this study, depend significantly on the quality of the curated chemical-gene expression association database. No algorithm would be able to identify the true CDT if no association between this CDT and the DE genes (or any gene) are annotated in the database used. Yet, the annotation of the drug-gene association database is the most challenging problem in the field. At any given time, these databases are incomplete, probably partially incorrect, and will evolve as the technology advances and more knowledge is gathered. However, the results shown here, demonstrate that the proposed approach will yield better results compared to the existing approaches when using currently available resources. The expectation is that an improvement of the quality of the underlying database will improve the results of all methods, rather than favor a particular one.

Moreover, all CDTs are not equally well studied. CDTs that are more popular and/or widely researched would have more associations with targeted genes discovered than the less popular ones. This issue, in turn, could create a potential bias against rare CDTs which would be less likely to be correctly identified. The same problem is observed in the pathway analysis field when the pathway analysis methods, including ORA, KS, Wilcoxon, and GSEA, tend to be biased toward small-size pathways^7^.

Another issue with the annotations is that any association can be recorded in the database in two different ways which will also affect the testing hypotheses. For example, let us consider a chemical C that increases the expression level of a gene G. This can be captured as either “C increases G” or, alternatively as “C deficiency decreases G”. In the experiment in which the chemical C is lacking (data set 14 in Table 2), instead of testing the hypothesis 2 that tests whether the chemical C’s level is lower than normal, one must test the hypothesis 1 with the true CDT being “chemical C deficiency”.

While benchmarking methods in terms of number of false positives reported, there could be similar CDTs that would have the same effects as the studied CDTs, and therefore, perhaps they should not be counted as false positives. However, in our opinion focusing on the exact chemical that was used to create the phenotype is the most objective and reproducible way for benchmarking the methods.

Finally, it is important to note that all methods compared here used public annotations from CTD with the exception of IPA which uses Qiagen’s proprietary knowledge base. In principle, the fact that IPA’s results were less accurate for some of the data sets could be due either to a lower quality underlying knowledge base or to an inferior algorithm. However, this distinction is less important in practical use. A life scientist contemplating the choice of the tools to use in identifying potentially causal CDTs could only consider IPA as a package including both knowledge base and associated algorithm. Therefore, we included the results obtained with IPA, as it is currently available to life scientists.

Although this topic is not new, public data sets related to this problem are scarce. To our knowledge, most of the published papers in this field include only one or two data sets in their manuscripts. For example, the causal analysis method in IPA only illustrated their method on two data sets in their manuscript. We considered that insufficient and we strived to use many more data sets. We did an exhaustive search but we only found the 16 data sets that we included here. This is still a small number of data sets but it is an order of magnitude more data sets than used in the articles presenting the existing methods in the field.

## Conclusion

The most important contribution of PURE is that it can infer the CDT responsible for the gene expression changes, which in turn causes the observed phenotype. This crucial ability is expected to be useful for the correct identification of the presence of chemicals, drugs or toxicants in new and unknown phenotypes. Moreover, PURE can identify the CDT that can revert disease-induced gene expression changes. This capability is expected to be useful in any drug repurposing application. In fact, this approach coupled with a pathway analysis, was able to repurpose methylprednisolone to treat severe symptoms related to hyper-inflammation of COVID-19 patients, very early in the pandemic, at a time when the WHO’s recommendation was against the use of steroids^41^.

The proposed approach was validated using 16 gene expression data sets from 3 different species where the true CDTs that caused the phenotypes were known. PURE correctly identified the CDT used in 11 out of 16 data sets (7 of which the true CDTs were ranked at the top). We also compared PURE to 5 other methods including a commercial tool, IPA. PURE outperformed all other methods in terms of the rank of the true CDT and the number of false positives in the list of significant CDTs.

## Data Availability

The raw data sets used in this study are listed in Table 2 and are downloaded from Gene Expression Omnibus (GEO: https://www.ncbi.nlm.nih.gov/geo/). The processed data is available on GitHub: https://github.com/gaminh/PURE.git.
