# Author Correction: A novel approach for predicting upstream regulators (PURE) that affect gene expression

**DOI:** 10.1038/s41598-024-64281-4

**Published:** 2024-06-10

**Authors:** Tuan‑Minh Nguyen, Douglas B. Craig, Duc Tran, Tin Nguyen, Sorin Draghici

**Affiliations:** 1 Department of Computer Science, Wayne State University, Detroit, 48202 USA; 2 Department of Oncology, School of Medicine, Wayne State University, Detroit, MI 48201 USA; 3 Department of Computer Science and Software Engineering, Auburn University, Auburn, 36849 USA; 4 Advaita Bioinformatics, Ann Arbor, MI 48105 USA; 5 Department of Medicine, Washington University School of Medicine, St. Louis, MO 63110 USA

Correction to: *Scientific Reports* 10.1038/s41598-023-41374-0, published online 30 October 2023

The Competing interests section in the original version of this Article was incorrect.

“The authors declare no competing interests.”

now reads:

“SD is the founder of Advaita Bioinformatics which is commercializing iPathwayGuide, which is an IPA alternative. The method proposed in this article is not commercialized by AdvaitaBio. No funding, salaries, equipment or supplies have been received from Advaita for this work. No consultation fees, in kind, or any other forms of remuneration were received for this work.”

The original Article has been corrected.

